# Antifibrotic effects of eupatilin on TGF-β1-treated human vocal fold fibroblasts

**DOI:** 10.1371/journal.pone.0249041

**Published:** 2021-03-25

**Authors:** Sung Joon Park, Hyunsu Choi, Ji Heon Kim, Choung-Soo Kim

**Affiliations:** 1 Department of Otorhinolaryngology-Head and Neck Surgery, College of Medicine, The Catholic University of Korea, Seoul, Republic of Korea; 2 Department of Otorhinolaryngology-Head and Neck Surgery, Yeouido St. Mary’s Hospital, College of Medicine, The Catholic University of Korea, Seoul, Republic of Korea; 3 Clinical Research Institute, Daejeon St. Mary’s Hospital, Daejeon, Republic of Korea; 4 Department of Otorhinolaryngology-Head and Neck Surgery, Daejeon St Mary’s Hospital, College of Medicine, The Catholic University of Korea, Daejeon, Republic of Korea; National Institutes of Health, UNITED STATES

## Abstract

Vocal fold scarring is a major cause of dysphonia. Vocal fold fibroblasts (VFFs) and the TGF-β signaling pathway play important roles in scar formation. Eupatilin, a chromone derivative of the *Artemisia* species, is a traditional folk remedy for wound healing. However, until recently, few studies investigated the therapeutic effects of eupatilin. We investigated the antifibrogenic effects of eupatilin on TGF-β1-treated human vocal fold fibroblasts (hVFFs). The optimal concentration of eupatilin was determined by a cell viability assay. Western blotting was used to measure the expression of alpha-smooth muscle actin during myofibroblast differentiation, fibronectin (FN), collagen type I (Col I), and collagen type III (Col III) extracellular matrix proteins, and Smad2, Smad3, and p38 in the fibrotic pathway. Measurements were made before and after eupatilin treatment. Eupatilin at 100 nM was shown to be safe for use in hVFFs. TGF-β1 induced hVFFs to proliferate and differentiate into myofibroblasts and increased Col III and FN synthesis in a time- and dose-dependent manner. Eupatilin suppressed TGF-β1-induced hVFF proliferation and differentiation into myofibroblasts through the Smad and p38 signaling pathways. Furthermore, eupatilin inhibited TGF-β1-induced FN, Col I, and Col III synthesis in hVFFs. Our *in vitro* findings show that eupatilin effectively suppressed TGF-β1-induced fibrotic changes in hVFFs via the Smad and p38 signaling pathways. Thus, eupatilin may be considered a novel therapeutic agent for the treatment of vocal fold fibrosis.

## Introduction

Chronic inflammation or severe injury to the vocal folds may induce fibrotic processes leading to vocal fold scarring, a major cause of dysphonia and impaired quality of life [1]. The transforming growth factor-beta (TGF-β) signaling pathway plays an important role in fibrogenesis in human vocal folds.

Previous studies have suggested that the TGF-β1 and Smad pathways are potential targets for the treatment of vocal fold scarring [2–4]. Several antifibrotic agents that target the TGF-β pathway have been investigated for their effect on fibrogenesis. Curcumin, a pharmacologically safe natural compound extracted from *Curcuma longa*, has been shown to suppress TGF-β1-induced cardiac fibroblast differentiation and reduce TGF-β1-induced expression of connective tissue growth factor in human gingival fibroblasts [5, 6]. Furthermore, retinoic acids, which are active vitamin A derivatives, have been shown to have an inhibitory effect on TGF-β1-induced expression of phosphorylated Smad in CCD-11Lu fibroblasts [7]. Nevertheless, several issues remain to be clarified, and efforts to identify a novel and effective antifibrotic agent that targets the TGF-β-mediated pathway continue.

During tissue fibrosis, TGF-β1 induces activity in both the canonical and non-canonical signaling pathways [8–10]. The binding of TGF-β1 to TGF-β receptor 2 recruits and activates TGF-β receptor 1; this results in the formation of the latent TGF-β complex [8], which activates the canonical pathway via the phosphorylation of Smad2, Smad3 [8, 9] and Smad-independent non-canonical pathways [8–10] such as the mitogen-activated protein kinase (MAPK) pathway [9, 10]. The MAPK family comprises three main kinases, including p38 [10], the phosphorylation of which in response to the latent TGF-β complex promotes MAPK pathway activation [8, 10]. Thus, the p38 MAPK pathway is a potential therapeutic target in pathologic fibrogenesis [9].

Eupatilin (5,7-dihydroxy-3,4,6-trimethoxyflavone) is a lipophilic polymethoxylated flavone isolated from *Artemisia asiatica*. Eupatilin has been reported to have antioxidative, anti-inflammatory, and anti-allergic effects, although the underlying mechanisms are unclear [11–14]. Moreover, eupatilin has been reported to have an anti-inflammatory effect on the TGF-β pathway [9].

We performed an *in vitro* study using human vocal fold fibroblasts (hVFFs) and hypothesized that eupatilin inhibits vocal fold fibrosis in humans by attenuating the TGF-β pathway. Fibronectin (FN), collagen type I (Col I), collagen type III (Col III), and alpha-smooth muscle actin (αSMA) were used as fibrotic markers. Furthermore, we identified the specific signaling pathways affected by eupatilin.

## Materials and methods

### Reagents

Dulbecco’s modified Eagle’s medium (DMEM), fetal bovine serum (FBS), and antibiotic-antimycotic solution were obtained from Gibco BRL (Gaithersburg, MD, USA). Recombinant human TGF-β1 was obtained from R&D Systems (Minneapolis, MN, USA). Eupatilin and p38 inhibitor (SB203580) were purchased from Sigma-Aldrich (St. Louis, MO, USA). Anti-FN, anti-Col I, anti-Col III, and anti- αSMA were purchased from Abcam (Cambridge, MA, USA). Anti-phosphorylated-Smad2/3 (anti-p-Smad2/3), anti-Smad2/3, anti-phosphrylated-p38 (anti-p-p38), anti-p38, anti-glyceraldehyde-3-phosphate dehydrogenase, and the secondary antibody (anti-rabbit IgG) were obtained from Cell Signaling Technology (Danvers, MA, USA). Detailed information on the antibodies is provided in S1 Table.

### hVFF cell culture

hVFFs were provided by Dr. Susan Thibeault (University of Wisconsin) [15, 16]. The cells were grown in DMEM (Gibco BRL) containing 2 mM L-glutamine, 100 mg/mL of penicillin-streptomycin, 2.5 mg/L of amphotericin B, and 10% heat-inactivated FBS (Gibco BRL). The cells were maintained at 37°C in a humidified atmosphere containing 5% CO_2_:95% air.

### Cell viability assay

A cell viability assay with five different concentrations of eupatilin (0, 100, 200, 500 and 1,000 nM) and three incubation times (24, 48, and 72 h) was performed to determine the optimal concentration and incubation duration of eupatilin. Cell viability was assessed using an Ez-Cytox cell viability assay kit (DoGen, Seoul, Republic of Korea) according to the recommended protocol. The kit reagent was added to each well, and the plates were incubated for 2 h at 37°C. The absorbance at 450 nm was measured using a microplate reader (Bio-Rad, Hercules, CA, USA).

### TGF-β1 treatment of hVFFs

Extracellular matrix (ECM) protein deposition and the myofibroblast differentiating potential of TGF-β1 in hVFFs were assessed by measuring the expression of FN, Col III, and αSMA (markers of myofibroblast differentiation) using Western blotting. Moreover, densitometric quantification was performed on hVFFs after incubation in 10 ng/mL of TGF-β1 for 24, 48, and 72 h. The dose-dependent effect of TGF-β1 was determined by incubating hVFFs in TGF-β1 (0, 1, 5, and 10 ng/mL) for 48 h. Then, we compared the Western blot and corresponding densitometric quantification findings in the treated and untreated fibroblasts. The phosphorylation-inducing potential of TGF-β1 on Smad2/3 and p38 in hVFFs was assessed by treating hVFFs with 10 ng/mL of TGF-β1 at three different time periods. We then used Western blotting and densitometric quantification to calculate the ratios of phosphorylated to nonphosphorylated Smad2/3 and p38.

### Assessment of cell proliferation and migration using the wound scratch assay

The cells were seeded into six-well plates. When the cell density reached >70%, scratch wounds were made by scraping the cell layer in each culture well using the tip of a 200 μL pipette. The cells were then washed with PBS and cultured in medium containing eupatilin for 48 h in the presence or absence of TGF-β1. After incubation, three fields were randomly chosen from each scratch wound and visualized under the microscope to assess cell migration. The experiments were performed in triplicate. The wound area was quantitatively determined using ImageJ software (National Institutes of Health, Bethesda, MD, USA). The percentage of the wound area was calculated as 100% minus the percentage of the denuded area/the initial scraped area.

### Western blotting

Cells were incubated in ice-cold RIPA lysis buffer (Elpis Biotech, Daejeon, Republic of Korea) containing protease inhibitor cocktail tablets (Roche Diagnostics, Mannheim, Germany). The lysates were centrifuged at 13,000 rpm for 15 min at 4°C. The supernatants were then collected and frozen at -80°C. Protein concentrations were determined using a BCA protein assay kit (Pierce, Rockford, IL, USA) according to the manufacturer’s instructions. The protein samples were separated by sodium dodecyl sulfate-polyacrylamide gel electrophoresis and transferred to nitrocellulose membranes (Bio-Rad), which were then probed overnight with primary antibodies. Next, the gels were incubated with horseradish peroxidase-conjugated secondary antibodies for 1 h at room temperature. The intensities of the signals were recorded and quantified using a molecular imaging system (Molecular Imager ChemiDoc XRS+; Bio-Rad).

### Statistical analyses

All of the experiments were repeated at least three times. Data are expressed as means ± SEM. All statistical tests were performed using Graph Pad Prism 5 software (GraphPad, Inc., La Jolla, CA, USA). Multiple group comparisons were made using a one-way analysis of variance followed by post-hoc Tukey’s tests. P-values <0.05 were considered to indicate statistical significance.

## Results

### Cytotoxicity and the optimal dose of eupatilin

**Fig 1:**
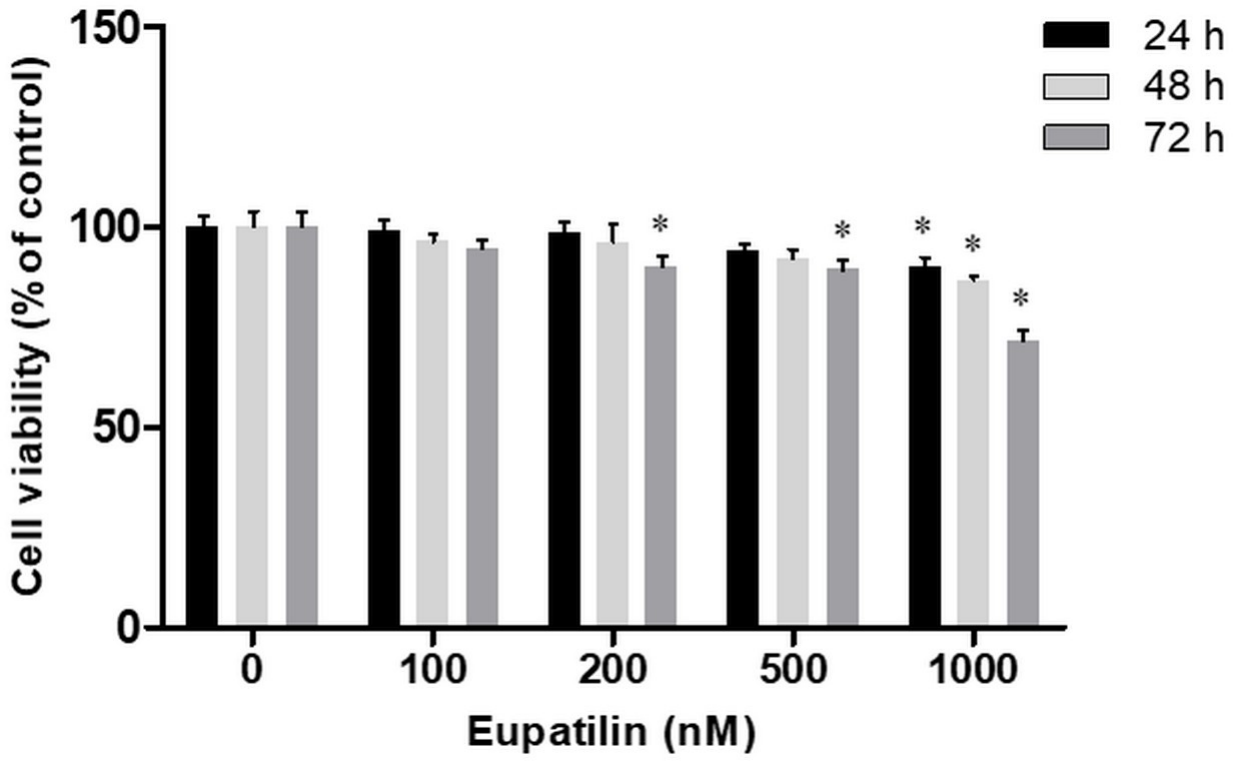
Effect of eupatilin on cytotoxicity in human vocal fold fibroblasts (hVFFs). The cells were incubated with eupatilin for the indicated times and at the indicated concentration. The viability of the hVFFs was then measured in a MTT assay. Data are representative of three independent experiments performed in triplicate and are expressed as the mean ± standard error of the mean (SEM). * p < 0.05 vs. untreated control (eupatilin = 0 nM).

A cell viability assay performed to assess the cytotoxic effect of eupatilin showed that after 72 h, the survival of hVFFs incubated in 200 nM and 500 nM eupatilin was significantly lower than that of hVFFs in the untreated control. Furthermore, 1,000 nM eupatilin had significantly fewer viable cells than the untreated control samples at all three incubation times In contrast, incubation in 100 nM eupatilin did not significantly affect cell viability at any incubation duration (Fig 1). Ultimately, 100 nM eupatilin was found to be safe for use in hVFFs.

### TGF-β1-induced ECM proteins in hVFFs

To determine the optimal dose of TGF-β1, hVFFs were treated with 0, 1, 5, and 10 ng/mL of TGF-β1 for 48 h. FN, Col III, and αSMA protein expression was detected by Western blotting. The expression of FN, Col III, and αSMA increased in a dose-dependent fashion with the addition of TGF-β1. Only 10 ng/mL of TGF-β1 significantly increased the expression of all three proteins compared to the untreated control. Therefore, 10 ng/mL was considered to be the optimal dose to induce fibrogenesis in hVFFs and was used in subsequent experiments (S1A and S1B File). To determine the optimal treatment duration, hVFFs were incubated in 10 ng/mL of TGF-β1 for 24, 48, and 72 h. Compared with the untreated controls, FN expression was significantly increased at all incubation durations. Furthermore, the expression of Col III and αSMA was significantly increased in hVFFs treated with TGF-β1 for 48 and 72 h. Therefore, our findings show that the application of 10 ng/mL of TGF-β1 for at least 48 h induced ECM protein expression and fibroblast differentiation into myofibroblasts *in vitro* (S1C and S1D File).

### TGF-β1-induced phosphorylation of Smad2 and p38 in hVFFs

We investigated the intracellular signaling pathways activated by 10 ng/mL of TGF-β1 in hVFFs. The phosphorylation of Smad2 and p38 increased significantly in hVFFs treated with 10 ng/mL of TGF-β for 48 h, which reflected activation of the Smad-based (Smad2) and non-Smad-based (p38 MAPK) signaling pathways. However, the levels of Smad2 and p38 phosphorylation were not significantly different between hVFFs incubated with TGF-β1 for 24 h and untreated controls (S2 File).

### Eupatilin suppressed TGF-β1-induced cell proliferation and migration in hVFFs

**Fig 2:**
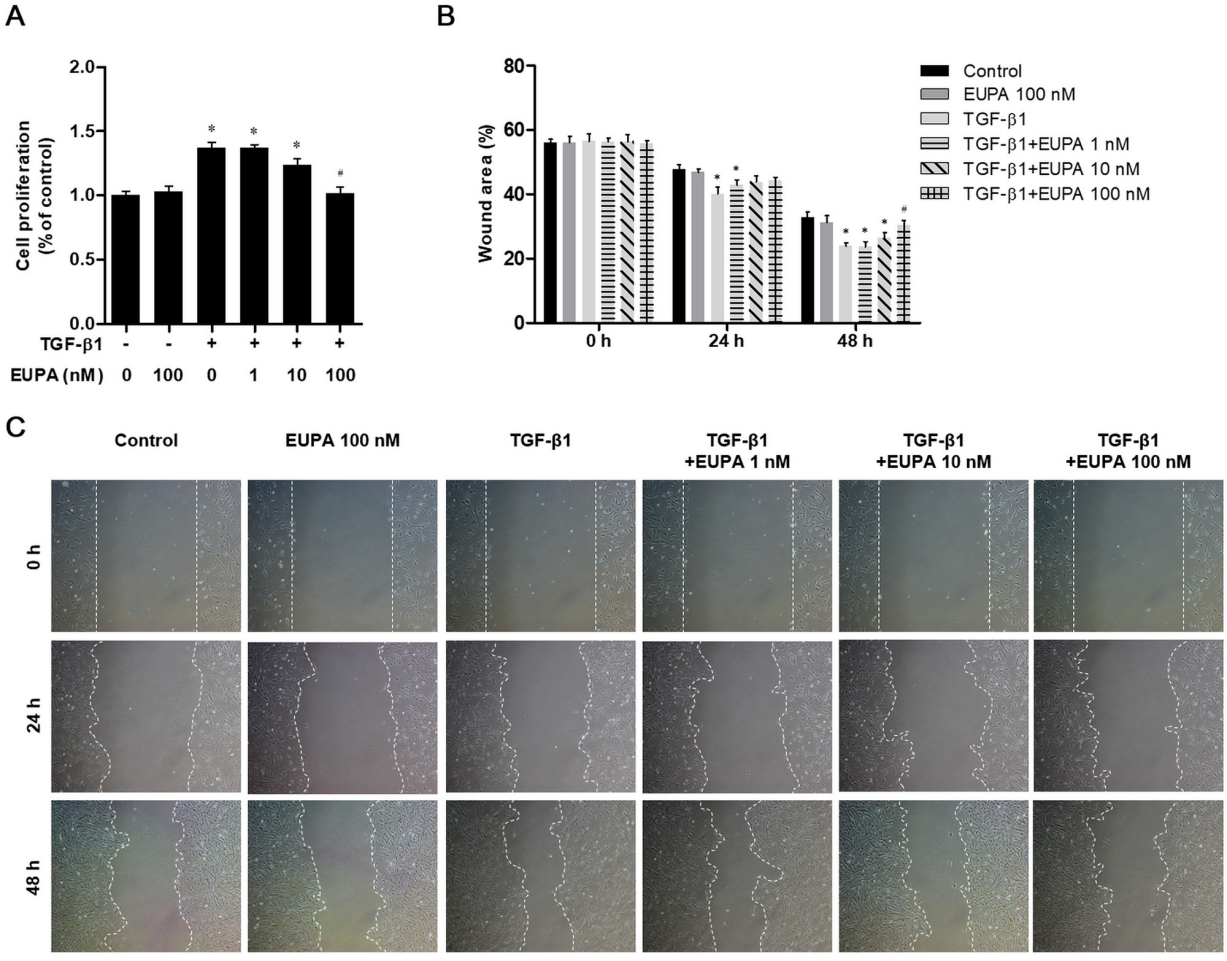
Effect of eupatilin on hVFFs proliferation and motility induced by TGF-β1. (A) hVFFs were treated for 48 h with TGF-β1 (10 ng/ml) plus 1, 10, or 100 nM eupatilin, or with 100 nM eupatilin alone. Cell proliferation was determined in an MTT assay. (B) Cell migration was evaluated by the wound healing assay. (C) Uncovered areas in the wound healing assays were quantified as the percentage of the original wound area. Data are representative of three independent experiments performed in triplicate and are expressed as the mean ± SEM. * p < 0.05 vs. TGF-β1 untreated control (TGF-β1 = 0 ng/ml).# p < 0.05 vs. eupatilin untreated control (eupatilin = 0 nM).

The proliferation of hVFFs treated with eupatilin (0, 1, 10, and 100 nM) in the presence of 10 ng/mL of TGF-β1 was compared with that of the untreated control cells. An MTT assay was performed to assess the inhibitory effect of eupatilin on TGF-β1-induced cell proliferation in hVFFs. Cell proliferation and wound area did not significantly differ from the untreated controls in hVFFs treated with 100 nM of eupatilin alone (Fig 2A). A wound scratch assay revealed that the denuded area was significantly smaller in the culture treated with 10 ng/mL of TGF-β1 alone compared with that of the untreated control. In cultures treated with 100 nM eupatilin, the denuded area was not significantly different from that of the untreated control culture after 48 h. In contrast, the denuded areas of the cultures treated with 1 and 10 nM eupatilin were significantly smaller than that in the untreated control cells, indicating that the lower eupatilin concentrations were not sufficient to inhibit the effect of TGF-β1 (Fig 2B and 2C).

### Eupatilin suppressed TGF-β1-induced fibrotic protein synthesis in hVFFs

**Fig 3:**
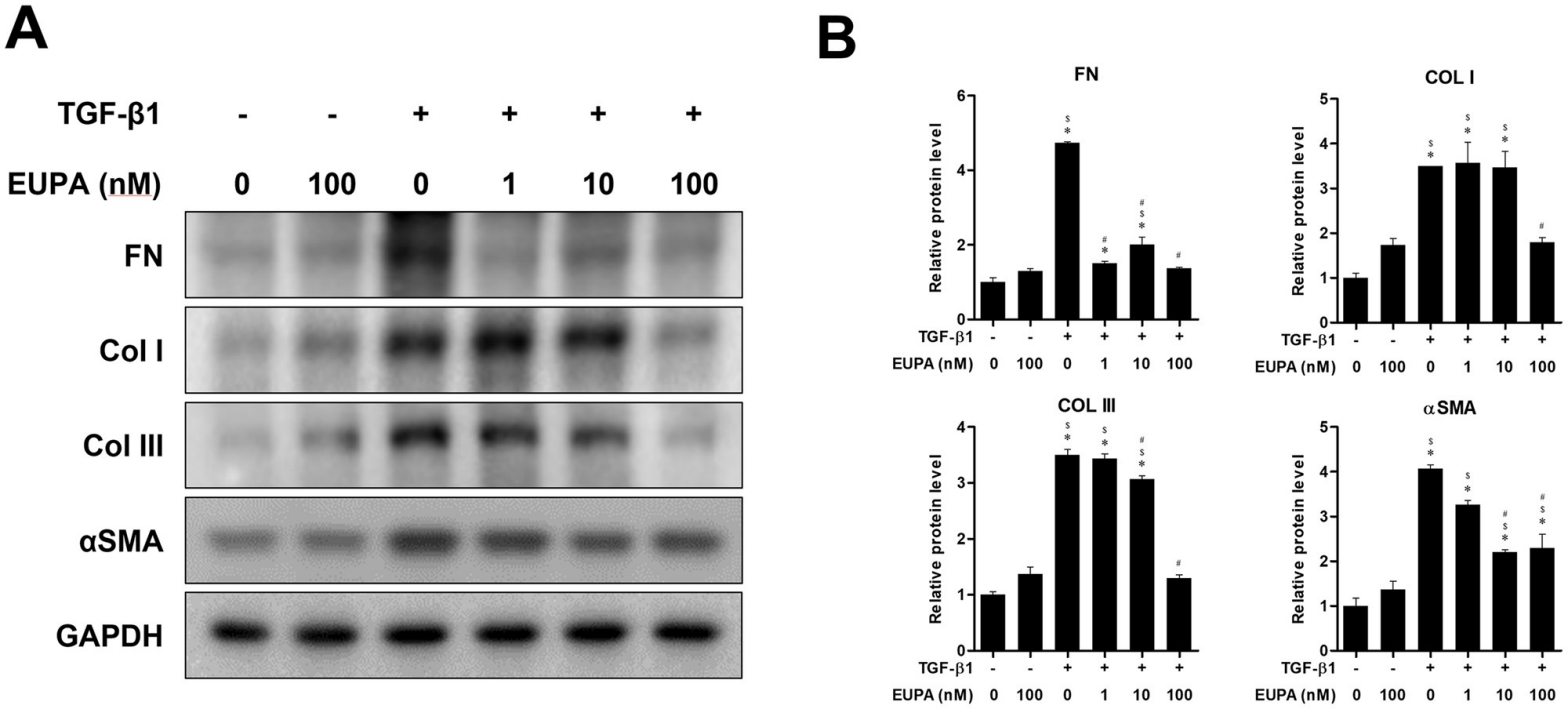
Effect of eupatilin on hVFF differentiation and TGF-β1-induced collagen deposition. (A) FN, COL I, Col III and αSMA protein levels in hVFFs treated with TGF-β1 (10 ng/ml) and eupatilin for 48 h. (B) Protein levels were quantified and then normalized based on GAPDH expression. Data are representative of three independent experiments performed in triplicate and are expressed as the mean ± SEM. * p < 0.05 vs. TGF-β1 untreated control (TGF-β1 = 0 ng/ml and eupatilin = 0 nM). # p < 0.05 vs. eupatilin untreated control (TGF-β1 = 10 ng/ml and eupatilin = 0 nM). $ p < 0.05 vs. eupatilin treated control (TGF-β1 = 0 ng/ml and eupatilin = 100 nM).

The inhibitory effects of 10 ng TGF-β1/mL on protein production were assessed in the presence of different concentrations of eupatilin and compared with untreated hVFFs and hVFFs treated with 100 nM eupatilin alone (Fig 3). Treatment of hVFFs with 1 nM eupatilin did not significantly suppress the TGF-β-induced synthesis of Col I, Col III, or αSMA. However, 10 nM eupatilin treatment significantly reduced the TGF-β-induced synthesis of FN Col III, and αSMA, although the protein levels of FN, Col I, Col III and αSMA remained significantly higher than in the TGF-β1 untreated controls. At 100 nM eupatilin, the TGF-β1-induced synthesis of FN, Col I, Col III, and αSMA was significantly lower than in the eupatilin untreated control.

### Eupatilin suppressed TGF-β1-induced Smad and p38 phosphorylation in hVFFs

**Fig 4:**
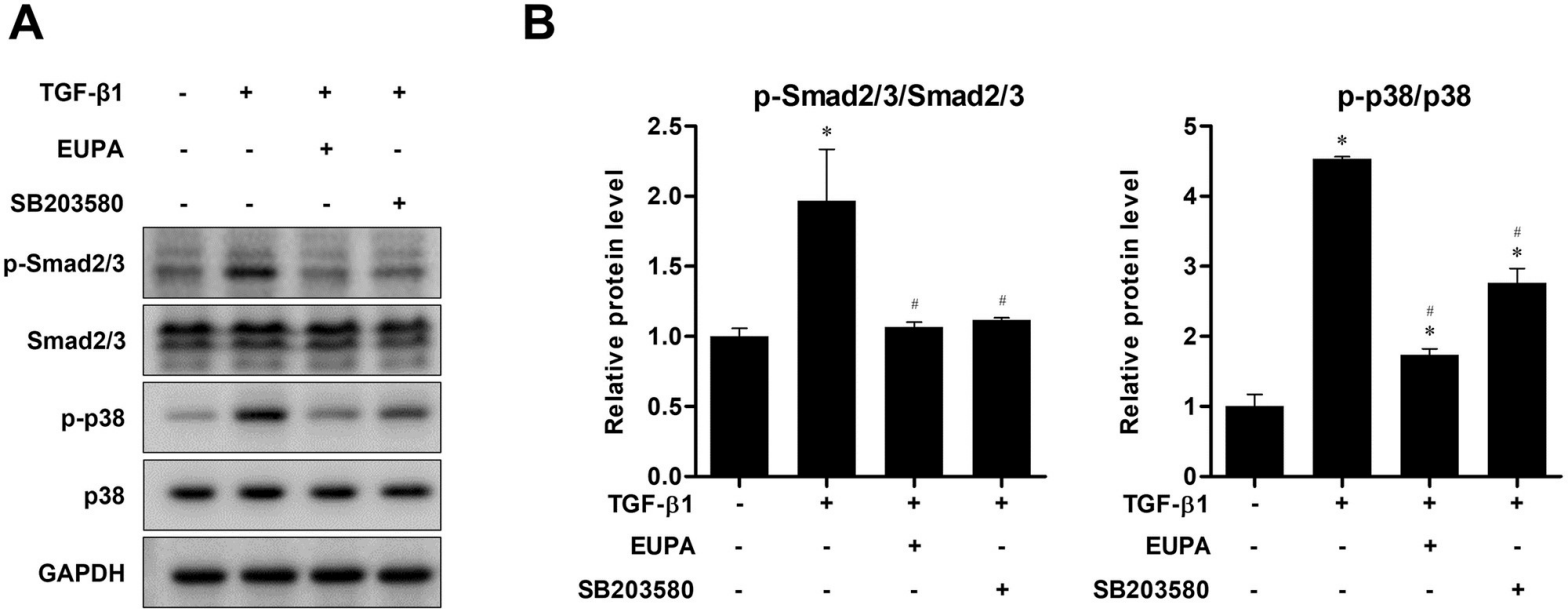
Effect of eupatilin on the TGF-β1-induced phosphorylation of Smad2/3, and p38 in hVFFs. (A) p-Smad2/3, and p-p38 protein levels in hVFFs treated with TGF-β1 (10 ng/ml) for 48 h in the presence or absence of SB203580 (10 μM) or eupatilin (100 nM). (B) Phosphorylation levels were quantified by densitometry and are presented as the ratio between the optical density of p-Smad2/3 and total Smad2/3, and p-p38 and total p38. Data are representative of three independent experiments performed in triplicate and are expressed as the mean ± SEM. * p < 0.05 vs. TGF-β1 untreated control (TGF-β1 = 0 ng/ml). # p < 0.05 vs. eupatilin untreated control (eupatilin = 0 nM).

The levels of Smad2/3, and p38 phosphorylation were determined by western blotting to investigate the effect of eupatilin on TGF-β1 signaling pathway activation in hVFFs. The treatment of hVFFs with 10 ng TGF-β1/mL significantly increased the phosphorylated proportion of Smad2/3, and p38. However, treatment with 100 nM eupatilin for 48 h significantly decreased the phosphorylation of Smad2/3 and p38 induced by 10 ng/mL of TGF-β1. Moreover, the phosphorylation levels of Smad2/3 was not significantly different from that in the untreated control cells. These findings suggest that eupatilin acts through both canonical (Smad-based) and noncanonical (non-Smad-based, p38) signaling pathways to inhibit the effect of TGF-β1 in hVFFs. Treatment with SB203580, a commercially available p38 inhibitor, for 48 h produced similar results to treatment with 100 μM eupatilin (Fig 4).

### Comparison of eupatilin and the p38 inhibitor on ECM protein expression and differentiation of hVFFs

**Fig 5:**
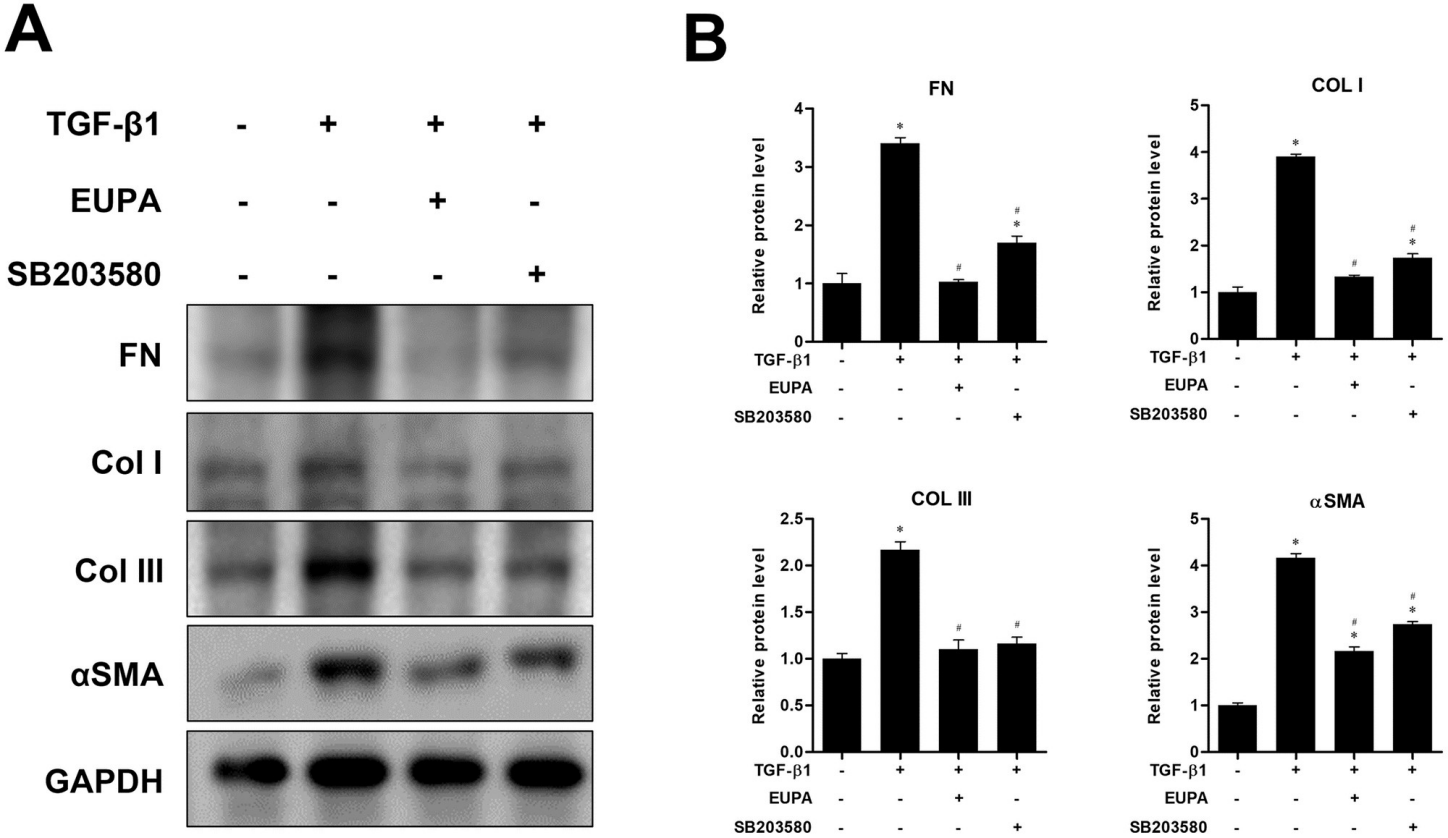
Effect of eupatilin and p38 inhibitor SB203580 on TGF-β1- induced hVFF differentiation and collagen deposition. (A) Protein levels of FN, Col I, Col III and αSMA in hVFFs treated for 48 h with TGF-β1 (10 ng/ml) in the presence or absence of SB203580 (10 μM) or eupatilin (100 nM). (B) Protein levels were quantified and then normalized based on GAPDH expression. Data are representative of three independent experiments performed in triplicate and are expressed as the mean ± SEM. * p < 0.05 vs. the TGF-β1 untreated control (TGF-β1 = 0 ng/ml and eupatilin = 0 nM). # p < 0.05 vs. untreated control (TGF-β1 = 10 ng/ml and eupatilin = 0 nM).

The inhibitory effect of eupatilin was assessed in terms of FN, Col I, Col III deposition, and αSMA expression induced by 10 ng/mL of TGF-β1. We compared the inhibitory effect of eupatilin with that of the p38 inhibitor SB203580. Treatment with 100 nM eupatilin and 10 μM SB203580 for 48 h significantly attenuated the TGF-β1-induced expression of FN, Col I, Col III, and αSMA. Moreover, the expression levels of FN, Col I and Col III in treatment with100 nM eupatilin were not significantly different from that of the TGF-β1 untreated control (Fig 5).

## Discussion

The main pathological feature of vocal fold scarring is disorganized composition of the ECM. Excessive collagen deposition and the accumulation of FN in the ECM are key pathological features of scarred vocal folds [17, 18]. Injury or inflammation of the vocal fold tissue activates fibroblasts, which differentiate into myofibroblasts to facilitate wound healing [19]. As a result, activated fibroblasts and myofibroblasts synthesize ECM proteins such as collagen, FN, and αSMA. The main modulator of these proteins is the TGF–β superfamily, particularly TGF–β1 [20–22].

TGF–β1 induced a dose-dependent increase in the production of FN, Collagen, and αSMA in hVFFs: incubation with 10 ng/mL of TGF–β1 for 48 h produced a significant increase in all three proteins. This finding is consistent with that of previous studies although in this study Col I and Col III production was analyzed in TGF–β1-treated hVFFs in the presence or absence of eupatilin. Col III seems to be more important than Col I in the early phase of wound healing, as it was expressed in the early phase of this process, followed by a gradual shift to Col I [19].

Smad2/3 and p38 phosphorylation increased significantly in hVFFs treated with 10 ng/mL of TGF–β1 for >48 h. Recent evidence suggests that TGF–β1 activates Smad-dependent signaling and Smad-independent signaling pathways (e.g., the MAPK pathway).

The binding of TGF–β1 to TGF–β receptor 2 induces the phosphorylation of Smad, activates TGF–β receptor 1 to form the latent TGF–β complex, and activates additional receptor signaling pathways [9]. Furthermore, TGF–β1 activates and regulates the Smad-independent signaling pathway (e.g., MAPK) [8]. p38 is one of three main kinases in the MAPK family [10].

Eupatilin has shown promise in inhibiting inflammation in both *in vitro* and *in vivo* models; however, the underlying mechanisms remain unclear [13]. In our study, eupatilin significantly inhibited TGF–β1-induced cell proliferation and hVFF migration without affecting cell viability. Moreover, eupatilin inhibited the differentiation of hVFFs into myofibroblasts and significantly decreased the TGF–β1-induced synthesis of Col I, Col III and FN in hVFFs. These finding are consistent with a recent study that found a potent antifibrotic effect of eupatilin in idiopathic pulmonary fibrosis [9].

Our findings show that eupatilin attenuated TGF–β1-induced phosphorylation of Smad2 and p38 in hVFFs. Moreover, the inhibitory effects of eupatilin and a p38 inhibitor, SB203580, on TGF–β1-induced fibrosis were not significantly different, suggesting that eupatilin affects TGF–β1-induced fibrogenesis via the modulation of TGF–β1 Smad-dependent and Smad-independent pathways. Several previous studies have shown that Smad2 and p38 are related to each other. Kamato et al. [23] showed that phosphorylation of the Smad linker regions is regulated by kinases such as p38.

SB203580, a specific p38 inhibitor, has been shown to downregulate TGF–β1-induced phosphorylation of the linker and C-terminal regions of Smad2 and Smad3 in rat mesangial cells [24]. Furthermore, Lee et al. [7] reported that a p38 MAPK inhibitor suppressed TGF-β1-induced p-Smad2 in a human fibroblast cell line (CCD-11Lu fibroblasts). Moreover, the findings of Hu et al. [25] suggest that SB203580 inhibits the phosphorylation of Smad2C, Smad2L and Smad3L in the human HCC HepG2 cell line. Our finding that SB203580 significantly suppressed TGF-β1-induced phosphorylation of Smad2, Smad3 and p38 in hVFFs is consistent with previous studies.

Although SB203580 showed promising results in our study, the p38-MAPK inhibitor is not suitable for clinical use due to several adverse effects. Xiao et al. [26] reported that SB203580 treatment enhanced hepatic lipoapoptosis in an *in vivo* rodent model of total parenteral nutrition and significantly promoted palmitic acid-mediated hepatic lipoapoptosis *in vitro*. Singh et al. [27] found that SB203580 was associated with significant activation of toxicity biomarkers, including increased phosphorylation of JNK in U937 cells and a marked increase in aspartate transaminase levels in HepG2 cells, suggesting significant hepatotoxicity *in vitro*. Moreover, previous studies of the anti-inflammatory effect of SB203580 have yielded inconsistent findings [28, 29]. Page et al. [30] reported that SB203580 significantly increased the lipopolysaccharide-stimulated pro-inflammatory cytokine interleukin (IL)-6 and decreased synthesis of the anti-inflammatory cytokine IL-10 in primary human monocytes.

Unlike SB203580, eupatilin has been approved for clinical use as an active ingredient in the commercially available herbal drug DA-9601 (Stillen^™^), which is used to treat gastric mucosal ulcers. Our study is the first to investigate the antifibrotic effect of eupatilin on vocal fold fibrosis, and our findings provide a basic understanding of the effectiveness and underlying molecular pathways of eupatilin in hVFFs. Given its known safety profile, our findings provide further support for eupatilin as a potential TGF–β-targeted antifibrotic agent for human vocal fold scarring.

Our study has several limitations. The formation of vocal fold scarring typically takes several months in humans. Because our study was limited to a 48-h period, we did not fully replicate the scarring in human vocal folds. Additional long-term studies are needed to validate our findings. Furthermore, *in vivo* studies are needed to confirm and extend our *in vitro* findings supporting eupatilin as a monotherapeutic agent targeting TGF-β1 pathways in vocal fold fibrosis.

## Conclusions

TGF–β1 treatment stimulated cell proliferation and induced expression of fibrotic ECM proteins in hVFFs. Eupatilin treatment inhibited cell proliferation and attenuated the expression of FN, Col I, Col III, and αSMA in TGF–β1-treated hVFFs via Smad and p38 pathways. Our findings have significant implications for TGF–β-targeted antifibrotic therapies for vocal fold scarring.

## Supporting information

S1 File Effect of TGF-β1 on hVFF differentiation and collagen deposition. (A, B) Dose-dependent changes in the expression of FN, Col III, and αSMA protein induced by incubation with TGF-β1 for 48 h, as measured by western blotting and adjusted for GAPDH expression. (C, D) Western blotting and corresponding densitometric quantification of FN, Col III, and αSMA expression in hVFFs treated with 10 ng TGF-β1/ml for the indicated times. Data are representative of three independent experiments performed in triplicate and are expressed as the mean ± SEM. * p < 0.05 vs. TGF-β1 untreated control (TGF-β1 = 0 ng/ml in A and B; 0 h in C and D).
(TIF)

S2 File Effect of TGF-β1 on the phosphorylation of Smad2 and p38 in hVFFs. (A) Effects on the phosphorylation of Smad2 and p38 in hvFFs incubated with TGF-β1 for the indicated times, as determined by western blotting. Total Smad2 and p38 served as the controls. (B) Phosphorylation levels were quantified by densitometry and are presented as the ratio between the optical density of p-Smad2 and total Smad2, and p-p38 and p38. Data are representative of three independent experiments performed in triplicate and are expressed as the mean ± SEM. * p < 0.05 vs. TGF-β1 untreated control (0 h).
(TIF)

S3 File (TXT)

S1 Fig Original blots corresponding to Fig 3A in the main text. (DOCX)

S2 Fig Original blots corresponding to Fig 4A in the main text. (DOCX)

S3 Fig Original blots corresponding to Fig 5A in the main text. (DOCX)

S1 Table List of antibodies used for western blot. (DOCX)
