# Supplementary figures and images for: Antifibrotic effects of eupatilin on TGF-β1-treated human vocal fold fibroblasts
Source: PLoS One. 2021 Mar 25;16(3):e0249041. doi: 10.1371/journal.pone.0249041 (PMC7993872; doi:10.1371/journal.pone.0249041)

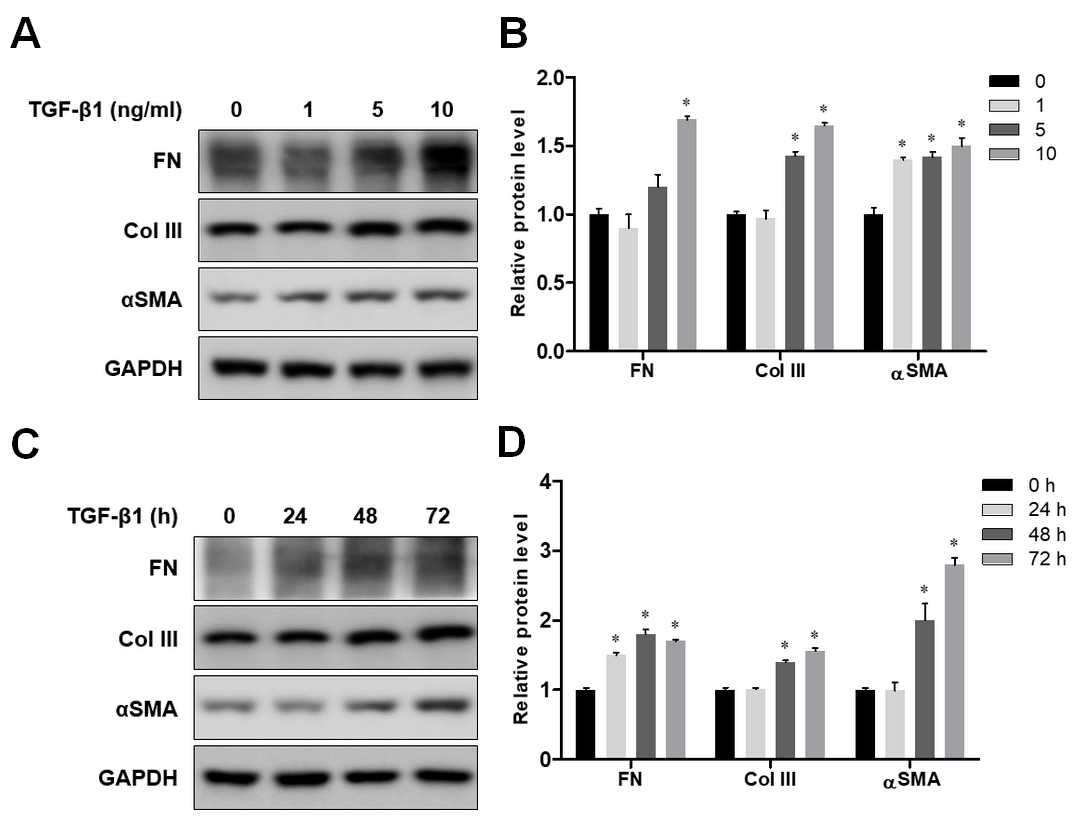

Supplement: S1 File — (A, B) Dose-dependent changes in the expression of FN, Col III, and αSMA protein induced by incubation with TGF-β1 for 48 h, as measured by western blotting and adjusted for GAPDH expression. (C, D) Western blotting and corresponding densitometric quantification of FN, Col III, and αSMA expression in hVFFs treated with 10 ng TGF-β1/ml for the indicated times. Data are representative of three independent experiments performed in triplicate and are expressed as the mean ± SEM. * p < 0.05 vs. TGF-β1 untreated control (TGF-β1 = 0 ng/ml in A and B; 0 h in C and D). (TIF) [file pone.0249041.s001.tif]

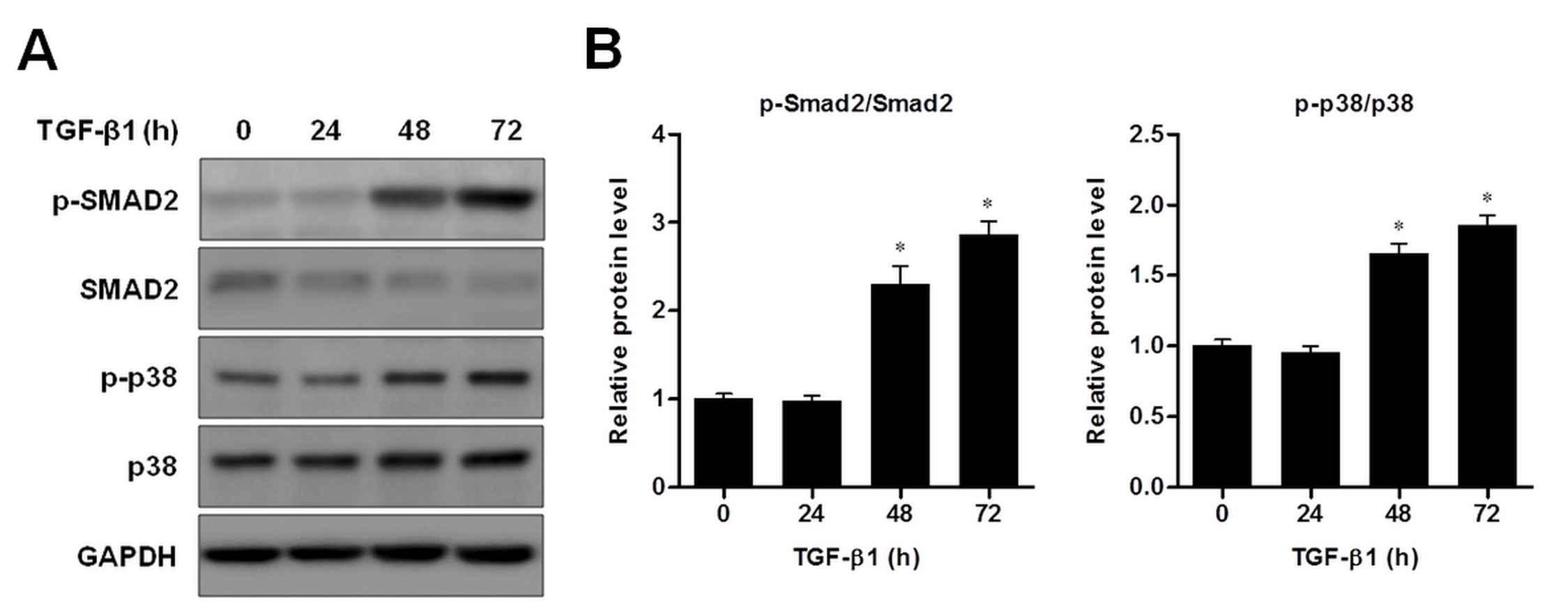

Supplement: S2 File — (A) Effects on the phosphorylation of Smad2 and p38 in hvFFs incubated with TGF-β1 for the indicated times, as determined by western blotting. Total Smad2 and p38 served as the controls. (B) Phosphorylation levels were quantified by densitometry and are presented as the ratio between the optical density of p-Smad2 and total Smad2, and p-p38 and p38. Data are representative of three independent experiments performed in triplicate and are expressed as the mean ± SEM. * p < 0.05 vs. TGF-β1 untreated control (0 h). (TIF) [file pone.0249041.s002.tif]

**S1 Fig.** Original blots corresponding to Fig.3A in the main text.


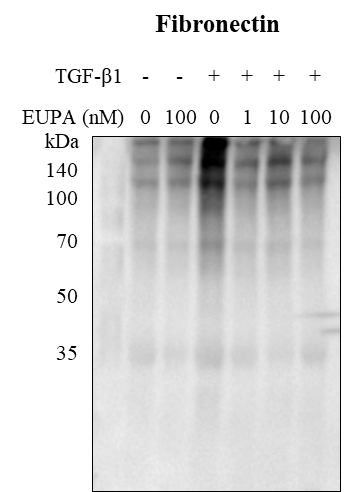

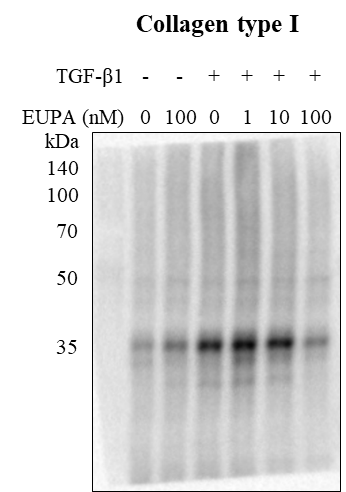


**
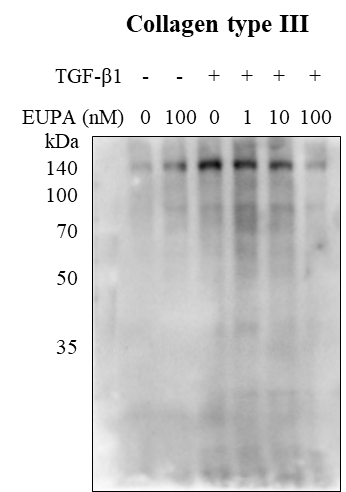

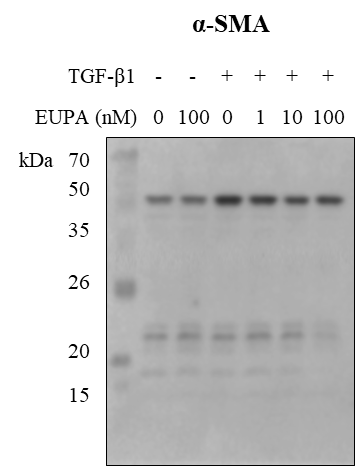
**

**
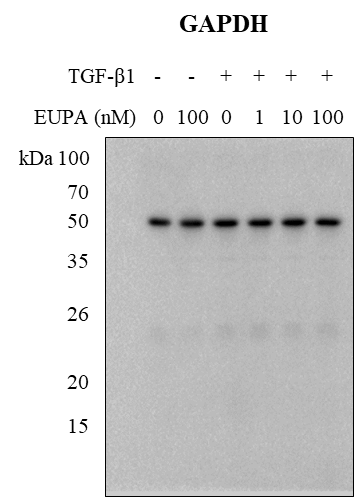
**

Supplement: S1 Fig — (DOCX) [file pone.0249041.s004.docx]

**S2 Fig.** Original blots corresponding to Fig.4A in the main text.


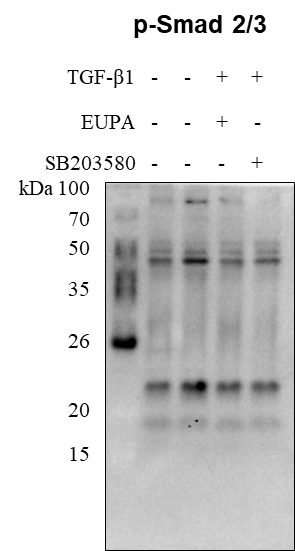

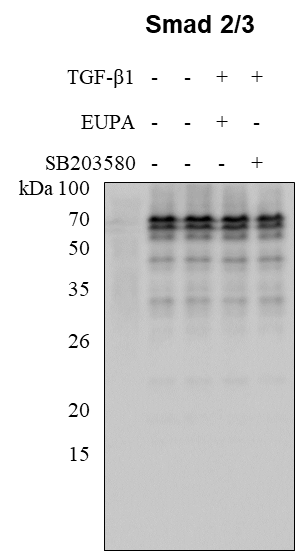


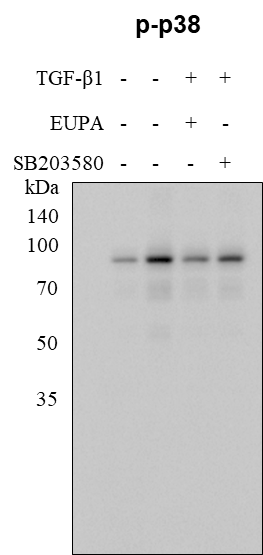

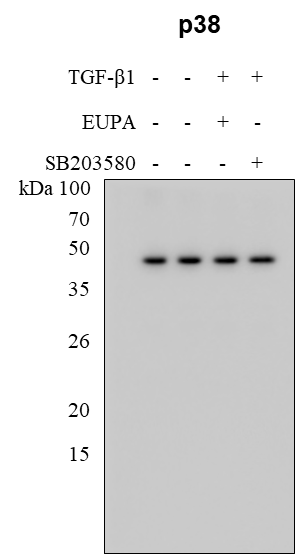


**
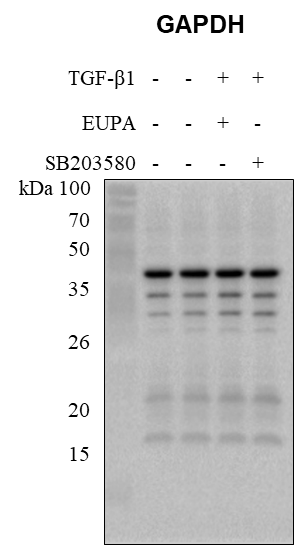
**

Supplement: S2 Fig — (DOCX) [file pone.0249041.s005.docx]

**S3 Fig.** Original blots corresponding to Fig.5A in the main text.


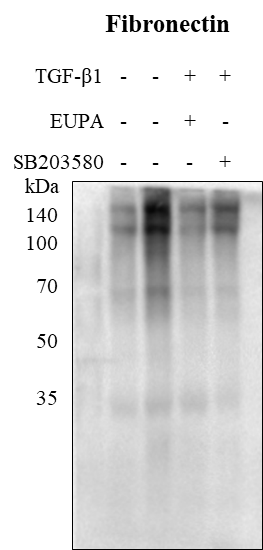

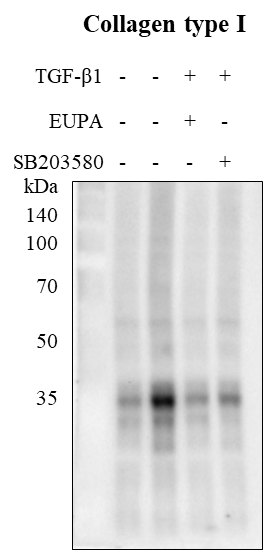


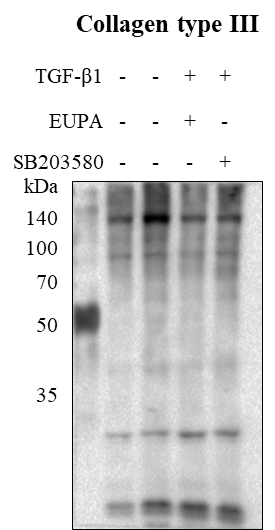

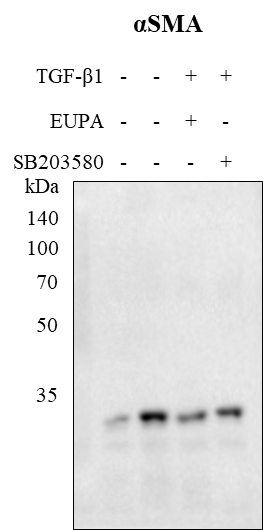

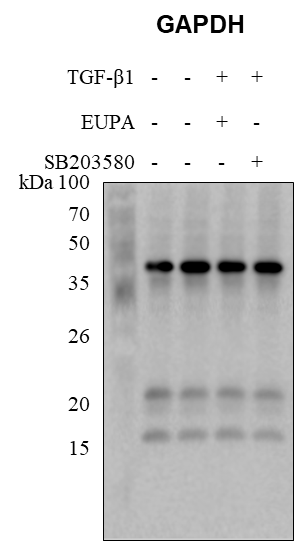

Supplement: S3 Fig — (DOCX) [file pone.0249041.s006.docx]
