# Supplementary material for: Antifibrotic effects of eupatilin on TGF-β1-treated human vocal fold fibroblasts
Source: PLoS One. 2021 Mar 25;16(3):e0249041. doi: 10.1371/journal.pone.0249041 (PMC7993872; doi:10.1371/journal.pone.0249041)
Supplement: S1 Table — (DOCX) [file pone.0249041.s007.docx]

**S1 Table. List of antibodies used for western blot.**

| **Primary antibodies** | **Supplier, Cat. No.** | **Species** | **Type** | **Dilution** |
| --- | --- | --- | --- | --- |
| Fibronectin | Abcam, ab2413 | Rabbit | polyclonal | 1 : 2000 |
| Collagen I | Abcam, ab255809 | Mouse | monoclonal | 1 : 1000 |
| Collagen III | Abcam, ab6310 | Mouse | monoclonal | 1 : 1000 |
| Alpha smooth muscle actin | Abcam, ab5694 | Rabbit | polyclonal | 1 : 1000 |
| Smad2/3 | Cell signaling, #3102 | Rabbit | polyclonal | 1 : 1000 |
| Phospho-Smad2 (Ser465/467)/Smad3  (Ser423/425) | Cell signaling, #8828 | Rabbit | monoclonal | 1 : 1000 |
| p38 MAPK | Cell signaling, #8690 | Rabbit | monoclonal | 1 : 1000 |
| Phospho-p38 MAPK | Cell signaling, #4511 | Rabbit | monoclonal | 1 : 1000 |
| GAPDH | Cell signaling, #5174 | Rabbit | monoclonal | 1 : 1000 |
